# Associations of Shift Work and Chronotype with Physical Activity and Eating Behaviors in Adult Women

**DOI:** 10.3390/nu18152507

**Published:** 2026-08-03

**Authors:** Nisanur Turan, Berrak Dumlupınar, Hasret Gülüş Oymak, Rabia Ela Ertem, Bekir Kürşat Aydın, İrem Karamollaoğlu, Arda Ozturkcan

**Affiliations:** 1 Department of Nutrition and Dietetics, Faculty of Health Sciences, İstanbul Okan University, 34959 Istanbul, Türkiye; nisaanurturaan@gmail.com (N.T.); hasret.oymak@okan.edu.tr (H.G.O.); rabia.ertem@okan.edu.tr (R.E.E.); kurrsat@gmail.com (B.K.A.); irem.karamollaoglu@okan.edu.tr (İ.K.); 2 Department of Nutrition and Dietetics, Faculty of Health Sciences, İstanbul Gelişim University, 34310 Istanbul, Türkiye; sozturkcan@gelisim.edu.tr

**Keywords:** obesity, emotional eating, shift work, chronotype, physical activity, dietary habits, women

## Abstract

**Background:** Shift work and chronotype may influence dietary habits, physical activity, and eating behaviors. However, research examining the combined associations of shift work and chronotype with these behaviors in women is limited. This study examined these associations, together with anthropometric outcomes, among women attending an obesity counseling center. **Methods:** A cross-sectional study included 190 women aged 18–64 years (mean age: 33.31 ± 7.87 years) attending an obesity counseling center in Istanbul. Sociodemographic data, dietary habits, work schedules and behavioral data were collected using structured questionnaires, whereas anthropometric measurements were obtained using standardized procedures. Chronotype, physical activity, and eating behaviors were assessed using the Morningness–Eveningness Questionnaire (MEQ), International Physical Activity Questionnaire (IPAQ), and Dutch Eating Behavior Questionnaire (DEBQ). Statistical analyses included chi-square tests, parametric-nonparametric group comparisons, and multivariable linear and logistic regression models. **Results:** Compared to fixed-schedule workers, shift workers had more frequent meals (77.9% vs. 59.6%; *p* = 0.007, V = 0.195) and breakfast (51.2% vs. 16.3%; *p* < 0.001,V = 0.371) skipping. Participants with obesity had higher emotional eating, external eating, total DEBQ, and total meal-number values than the other BMI groups. Chronotype scores varied according to age, smoking, alcohol consumption and occupational factors, but were not directly correlated with anthropometric measurements. **Conclusions:** Shift work was associated with irregular eating habits and altered eating behaviors. These findings should be interpreted with caution because the cross-sectional, single-center design limits causal inference and generalizability. To better understand the relationship between chronotype, lifestyle, and obesity-related behaviors, as well as to develop effective interventions, larger prospective studies are needed.

## 1. Introduction

The irregular work schedules that are characteristic of modern life, particularly those involving shift work, disrupt human circadian rhythms, resulting in substantial alterations to sleep patterns and eating habits. Chronotype is a concept that reflects an individual’s circadian rhythms, thereby demonstrating differences in the timing of physiological processes such as body temperature, cortisol, and melatonin. These differences determine the hours during which individuals exhibit high levels of attention and work efficiency, and influence eating behaviors [1]. Individuals who are active late in the evening tend to consume more energy from animal protein, alcohol, processed sugars, and fats, while those who wake up early in the morning tend to consume more vegetables, legumes, calcium, and vitamin B6 [2]. A substantial relationship has been identified between nocturnal sleep patterns and the consumption of caffeine and processed foodstuffs. The impact of shift work on eating habits and metabolic responses has been a subject of considerable research. Human metabolism is not well-suited to nocturnal eating; the consumption of food at late hours increases the LDL/HDL cholesterol ratio and raises plasma triglyceride levels, increasing the risk of cardiovascular disease [3]. The literature suggests that lipid profiles are elevated in individuals who engage in shift work. Furthermore, sleep disorders are prevalent among individuals engaged in shift work, a phenomenon attributable to the inability of the circadian rhythm to adapt adequately. This, in turn, may contribute to impaired metabolism and an increased risk of mortality over time [4].

Moreover, epidemiological studies have indicated an elevated risk of specific types of cancer, including breast, colon, prostate, and endometrial cancer, in individuals who work night shifts. The International Agency for Research on Cancer has identified shift work as a “possible carcinogenic risk factor”. A meta-analysis by Wang et al. reported that the risk of colorectal cancer in individuals engaged in night-time work increased by 11% for every five-year period in comparison with those engaged in day-time work [5,6,7].

A number of studies have demonstrated an association between chronotype and sleep status, physical activity, and food intake. Meta-analyses demonstrate that a reduced duration of sleep leads to an increase in total daily energy intake in healthy adults. Dashti et al. [8] summarized evidence that individuals who sleep for short periods consume high-calorie meals and snacks at irregular intervals, thus revealing a relationship between insufficient sleep and binge eating. As demonstrated by Markwald et al. [9], in subjects whose habitual sleep duration was reduced to 5 h for a period of 5 days, whilst there was a 5% increase in energy expenditure, the increase in food intake was more pronounced. This finding suggests that insufficient sleep duration can result in a positive energy balance and moderate weight gain. The ingestion of high glycemic index carbohydrates during nocturnal hours has been demonstrated to engender a state of somnolence and diminish cognitive function when compared with the consumption of high-fat diets [8,9,10,11,12,13].

It is evident that irregular working hours, chronic stress, low socioeconomic status, and unhealthy eating habits disrupt the circadian rhythm, with the result that there is a negative effect on energy balance, glucose metabolism, and appetite control. These factors, it can be argued, are risk factors for the development of obesity. Furthermore, fluctuations in individuals’ emotional states (excitement, happiness, sadness, fear, anxiety) have been demonstrated to influence their food preferences and the quantity and frequency of their meals [14,15,16,17].

Morning-evening chronotype differences have been demonstrated to impact eating habits and attitudes, and therefore it is important to investigate this relationship. Evidence suggests that individuals with a short sleep duration exhibit elevated insulin and fasting blood glucose levels. The disruption of sleep patterns, consequent to shift work, has been demonstrated to result in alterations to meal times, frequency, energy intake, and eating behaviors. The consumption of high-saturated fat and simple carbohydrates, increased meal frequency, high caffeine and alcohol use, and vitamin–mineral deficiencies have been demonstrated to have a detrimental effect on health. The phenomenon of insomnia, precipitated by shift work, has been shown to result in an elevation of ghrelin and a concomitant decrease in leptin. This hormonal imbalance has been demonstrated to engender a disruption in appetite regulation, thereby contributing to the exacerbation of weight issues. Furthermore, it has been demonstrated that shift work increases simple carbohydrate intake while decreasing fiber intake, leading to irregular meal times. Conversely, sleep deprivation has been demonstrated to increase total energy intake and food consumption [18,19,20,21,22].

The impact of intense work schedules and variable working hours on health behaviors, particularly dietary habits, is a subject that has been the focus of numerous studies. The phenomenon of emotional eating, precipitated by stress and negative emotions, has been demonstrated to be associated with working conditions and individual chronotypes. Therefore, the primary aim of this study was to compare women working shift and fixed schedules in terms of lifestyle characteristics, dietary habits, physical activity levels, chronotype, and emotional, restrained, and external eating behaviors. A secondary aim was to explore the relationships among work schedule, chronotype, anthropometric measurements, physical activity, and eating behaviors. We hypothesized that shift work and evening chronotype would be associated with more irregular eating patterns, lower physical activity levels, and higher scores of maladaptive eating behaviors.

## 2. Materials and Methods

### 2.1. Study Design and Participants

This cross-sectional study was conducted between November 2024 and January 2025 at an obesity counseling center located in the Maltepe district of Istanbul. The current study comprised a total of 190 female participants aged 18 to 64 years, working in either shift or fixed schedules. The scoring and evaluation of the subscales were developed by examining the questionnaires and taking into account the 57 participants with chronic illnesses. The exclusion criteria encompassed males, individuals below the age of 18 or above 65 years of age, pregnancy or lactation period, individuals not willing to participate, and those following special diet programs.

### 2.2. Sample Selection

The population and sample size of the study were determined by power analysis performed using G*Power 3.1.9.2 software. The analysis was conducted using the *t*-test for independent samples at a 95% confidence level. The requisite sample size for the study was determined by calculating the power (1 − β) of the study (1 − β) 0.90, alpha type error 0.05, beta type error 0.10 and effect size 0.48, utilizing the findings of Arslan’s et al. analogous research, and the minimum sample size was thus established as 186. In order to account for the potential loss of data, it was decided that the study would be completed with a total of 190 participants [23].

### 2.3. Data Collection

The administration of a questionnaire in a clinical setting served as the primary data collection instrument. The questionnaire was administered to 190 volunteer individuals. The questionnaire was divided into three sections. The questionnaire method was utilized to obtain data regarding demographic characteristics, work arrangements, anthropometric measurements and general eating habits. Information was also collected from individuals using one-day 24 h dietary recall and 29-item Food Frequency Questionnaire (FFQ) and the Physical Activity Questionnaire (IPAQ). In addition to the general questionnaire, participants were asked to complete the Morningness–Eveningness Questionnaire (MEQ) in order to assess their chronotype, and the Dutch Eating Behavior Questionnaire (DEBQ) in order to determine their eating attitudes and behaviors.

The nature of the research is such that it is based on information provided by the participants. Consequently, the information is subjective in nature. Consequently, the research data is constrained due to the potential for bias.

#### 2.3.1. Dietary Assessment

The assessment of dietary intake was conducted through the utilization of a one-day 24 h dietary recall method, which was administered via a questionnaire. Furthermore, a 29-item FFQ encompassing major food groups was utilized to evaluate the frequency of food consumption. Nutrient intake analysis was performed using the computer-assisted Nutrition Information System Software (BeBiS 9) to calculate energy and nutrient intakes from the dietary records. The FFQ incorporated seven consumption frequency categories, ranging from daily to never. These methods are standard tools that are frequently utilized in nutritional assessment studies.

#### 2.3.2. Physical Activity Assessment

The physical activity levels of the participants were evaluated using the International Physical Activity Questionnaire (IPAQ), which was developed in 1998 based on a standardized international framework. The IPAQ is a tool designed to assess the duration and frequency of physical activities performed during the previous seven days. These activities include walking, moderate-intensity activity, vigorous-intensity activity, and sitting time.

The total physical activity score was calculated as metabolic equivalent task minutes per week (MET-min/week) by multiplying the duration (minutes), frequency (days), and corresponding MET values for each activity category. The MET values were defined as 3.3 for walking, 4.0 for moderate-intensity physical activity, and 8.0 for vigorous-intensity physical activity. Participants were classified according to physical activity level categories, with the classification being based on the calculated MET-min/week scores. The Turkish validity and reliability study of the IPAQ was conducted by Ozturk. The IPAQ is a widely utilized instrument for the standardized assessment of physical activity levels in epidemiological and nutritional studies [24].

#### 2.3.3. Morningness–Eveningness Questionnaire (MEQ)

The chronotype characteristics of the participants were assessed using the Morningness–Eveningness Questionnaire (MEQ), which was originally developed by Horne and Ostberg in 1976 to determine individual chronotype preferences [25]. The questionnaire under consideration consists of 19 multiple-choice items, the purpose of which is to evaluate preferred sleep–wake timing, as well as the periods during which individuals perceive their psychological and physical performance to be optimal throughout the day. The Turkish validity and reliability study of the MEQ was conducted by Pündük et al. [26].

The MEQ employs a Likert-type scoring system with structured response categories. Responses to questions 1, 2, and 10 are arranged using a time scale covering a seven-hour interval divided into 15 min periods, while the remaining items are presented as closed-ended response options. According to the selected responses, participants receive scores ranging from 1 to 4 for Questions 3–9 and 13–16, from 1 to 5 for Questions 1, 2, 10, 17, and 18, from 0 to 6 for Questions 11 and 19, and from 0 to 5 for Question 12. The total MEQ score ranges from 16 to 86 points. Participants were classified as morning type (59–86 points), intermediate type (42–58 points), or evening type (16–41 points) based on the total score. The MEQ is a widely utilized and validated instrument for the assessment of chronotype in epidemiological and clinical studies [27].

#### 2.3.4. Dutch Eating Behavior Questionnaire (DEBQ)

The Dutch Eating Behavior Questionnaire (DEBQ) was developed by Van Strien et al. [28] in 1986 with the purpose of assessing different dimensions of eating behavior. The scale comprises 33 items and evaluates three sub-dimensions of eating behavior: emotional eating (e.g., “Do you have a desire to eat when you are irritated?”), external eating (e.g., “Do you eat when you see or smell delicious food?”), and restrained eating (e.g., “Do you try to eat less the day after overeating?”). Responses are evaluated using a five-point Likert scale, ranging from 1 (never) to 5 (very often). It is important to note that each subscale is evaluated independently, and there is no overall cutoff score. It is evident that higher scores are indicative of more pronounced maladaptive eating behavior patterns. The Turkish validity and reliability study of the DEBQ was conducted by Bozan, and the scale was adapted into Turkish in 2009. In the Turkish version of the scale, items 1–10 assess restrained eating behavior, items 11–23 assess emotional eating, and items 24–33 assess external eating. Item 31 in the external eating subscale is reverse scored [28,29].

#### 2.3.5. Anthropometric Measurements

The researcher took the height and weight measurements of the participants according to standardized procedures. Participants were measured without shoes and wearing light clothing, standing upright and looking straight ahead with the Frankfort Plane (the line from the upper part of the ear to the outer corner of the eye) parallel to the floor. The height of the participants were measured using a calibrated digital stadiometer (Nan DR-MOD-85, Istanbul, Turkey), respectively, with a capacity of 10–200 kg and 90–200 cm, and a precision of ±100 g and ±1 mm and body weight was measured using a bioelectrical impedance analysis device (Tanita MC-780; Tanita Corporation, Tokyo, Japan), which possesses a capacity of 270 kg and an accuracy of 100 g. The body mass index (BMI) was calculated using the following formula: weight (in kilograms) ÷ height (in meters squared). Waist and hip circumferences were measured with a non-stretchable tape measure while the participants stood with arms at their sides and feet parallel, maintaining balance on each leg. These anthropometric measurements were undertaken in accordance with standardized protocols, ensuring the accuracy and reliability of the data obtained.

### 2.4. Statistical Analysis

Statistical analyses were performed using IBM SPSS Statistics software (version 26.0; IBM Corp., Armonk, NY, USA). The normality of data distribution was assessed using the Kolmogorov–Smirnov test, given that the sample size was greater than 30. The distribution of continuous variables was assessed using skewness and kurtosis coefficients. Normally distributed variables are presented as mean ± standard deviation (SD), whereas non-normally distributed variables are presented as median (Q1–Q3). For two-group comparisons, the independent-samples *t*-test, with Welch correction when required, or Mann–Whitney U test was used as appropriate. For comparisons across three or more groups, one-way ANOVA or Kruskal–Wallis test was used as appropriate. Post hoc analyses were performed only when the overall multi-group test was statistically significant. Bonferroni-adjusted pairwise comparisons were used after ANOVA, Dunn–Bonferroni pairwise comparisons after Kruskal–Wallis tests, and Bonferroni-adjusted pairwise comparisons of column proportions were used for significant categorical variables. Categorical variables were analyzed using the Pearson chi-square test. A two-sided *p* value of <0.05 was considered statistically significant. Effect sizes were reported for the primary group comparisons and associations irrespective of statistical significance; Cohen’s d was reported for independent-sample *t*-tests, rank-biserial r for Mann–Whitney U tests, eta-squared (η^2^) for one-way ANOVA, epsilon-squared (ε^2^) for Kruskal–Wallis tests, and Cramer’s V for chi-square analyses.

For regression analyses, candidate variables were first identified for each dependent variable using univariable analyses at *p* < 0.05. Only variables that remained statistically significant at *p* < 0.05 in the final multivariable model were retained. Logistic regression results are presented as odds ratios (OR) and 95% confidence intervals (CI), whereas linear regression results are presented as unstandardized coefficients (B), standardized beta coefficients (β), and 95% CI. Physical activity was calculated as MET-min/week according to the IPAQ protocol, using MET coefficients of 3.3, 4.0, and 8.0 for walking, moderate-intensity activity, and vigorous-intensity activity, respectively. Chronic disease status was included in group comparisons and considered as a candidate predictor in multivariable regression models when it was significant in univariable screening.

## 3. Results

### 3.1. General Characteristics of the Participants

The study included a total of 190 adult women with a mean age of 33.31 ± 7.87 years, indicating that the sample was predominantly composed of young and middle-aged adults. Slightly more than half of the participants were single, and the majority had a university degree or higher. In terms of occupational distribution, healthcare workers and private sector employees constituted the largest groups. Fixed working hours were more common than shift work hours, although a significant proportion of participants reported working shifts. More than half of the participants reported smoking, and alcohol consumption varied across the sample. The mean BMI of the participants was 25.20 ± 4.03 kg/m^2^, corresponding to the mean overweight range. According to BMI classification, more than half of the participants were in the normal weight range, while the remainder were classified as overweight or obese. Participants generally showed moderate chronotype characteristics, and eating behavior scores pointed out measurable tendencies toward restrictive, emotional, and external eating.

### 3.2. Characteristics According to Work Schedule

In categorical analyses, the proportion of married participants was higher in the fixed-schedule group, whereas the proportions of participants with university or higher education and healthcare workers were higher in the shift work group (all *p* < 0.05). Presence of chronic disease, smoking, and physical inactivity did not differ significantly according to work schedule (all *p* > 0.05). Meal skipping was more frequent among shift workers than fixed-schedule workers (77.9% vs. 59.6%; *p* = 0.007). Breakfast skipping was also significantly more frequent among shift workers (51.2% vs. 16.3%; *p* < 0.001).

Age differed significantly according to work schedule, with a lower mean age in shift workers than in fixed-schedule workers (30.85 ± 5.66 years vs. 35.34 ± 8.84 years; *p* < 0.001; d = 0.593).

Among anthropometric variables, hip circumference was significantly lower in shift workers than in fixed-schedule workers (97.03 ± 9.22 cm vs. 100.31 ± 7.48 cm; *p* = 0.009; d = 0.394). Waist circumference was lower in shift workers than in fixed-schedule workers (78.83 ± 10.64 cm vs. 81.76 ± 9.82 cm; *p* = 0.050; d = 0.288), whereas BMI did not differ significantly by work schedule (*p* = 0.126). MEQ score was significantly lower in shift workers than in fixed-schedule workers (44.92 ± 11.24 vs. 55.74 ± 10.11; *p* < 0.001; d = 1.017) (Table 1). Among DEBQ subscales, external eating score was significantly higher in shift workers (32.26 ± 4.19 vs. 25.92 ± 5.95; *p* < 0.001; d = 1.211), and the total DEBQ score was also higher in shift workers (92.06 ± 15.30 vs. 86.37 ± 20.96; *p* = 0.032; d = 0.306). DEBQ emotional eating score did not differ significantly between work schedule groups (*p* = 0.712). Among the categorical variables, marital status, university or higher education level, being a healthcare worker, skipping meals and breakfast showed significant differences according to work schedule. The largest categorical difference was observed in the healthcare worker group (V = 0.552), followed by skipping breakfast (V = 0.371), university or higher education level (V = 0.262), skipping meals (V = 0.195), and marital status (V = 0.143). The presence of chronic disease, smoking, physical inactivity, and BMI ≥ 25 did not show a significant difference according to work schedule.

### 3.3. Characteristics According to Chronotype

Age differed significantly among chronotype groups (*p* < 0.001; η^2^ = 0.090). Bonferroni post hoc analysis showed that the mean age of the morning type group was significantly higher than both the mid-type and evening type groups (*p* = 0.002 and *p* = 0.001, respectively), while no significant difference was found between the mid-type and evening type groups (*p* = 1.000). Body weight and BMI did not show a significant difference among chronotype groups (*p* = 0.186 and *p* = 0.126, respectively). Hip circumference, however, differed significantly according to chronotype classification (*p* = 0.004; η^2^ = 0.058). Bonferroni-corrected pairwise comparisons showed that hip circumference was higher in the morning chronotype group compared to the intermediate chronotype group (*p* = 0.002), whereas comparisons between morning and evening chronotype, and between and evening chronotype, were not statistically significant (*p* = 0.632 and *p* = 0.227, respectively). Therefore, the anthropometric results do not support the statement that evening chronotype is associated with higher body weight, BMI, or hip circumference in this sample.

IPAQ MET-min/week differed significantly across chronotype groups (*p* = 0.013; η^2^ = 0.045); Bonferroni post hoc analysis showed that the intermediate-type group had significantly higher MET-min/week values than the evening-type group (*p* = 0.020). On the other hand, morning type vs. intermediate type and morning type vs. evening type comparisons were not significant (*p* = 0.158 and *p* = 1.000, respectively).

DEBQ restrained eating, emotional eating, external eating, and total scores all differed significantly across chronotype classes (all *p* < 0.05). DEBQ restricted eating, emotional eating, and total DEBQ scores were analyzed using one-way ANOVA; therefore, pairwise results are based on Bonferroni-corrected post hoc comparisons. Restricted eating was found to be higher in the evening type group compared to both the morning and intermediate-type groups (*p* < 0.001 and *p* = 0.002, respectively; η^2^ = 0.103). The total DEBQ score was higher in the evening-type group than in both morning-type and intermediate-type groups (*p* < 0.001 for both comparisons; η^2^ = 0.146). Emotional eating scores were found to be higher in the evening eating group compared to the morning and mid-morning eating groups (*p* = 0.002 and *p* = 0.005, respectively; η^2^ = 0.085). DEBQ external eating behavior was assessed using one-way ANOVA; Bonferroni post hoc analysis revealed that both the mid-morning and evening eating groups had higher scores than the morning eating group (*p* = 0.032 and *p* = 0.017, respectively; η^2^ = 0.056).

The total number of meals differed significantly among chronotype groups (*p* = 0.017; η^2^ = 0.043), and significant pairwise differences were observed between morning and intermediate-type groups and between evening and intermediate-type groups. Among categorical variables, shift work, chronic disease, skipping meals, skipping breakfast, and BMI ≥ 25 showed significant differences among chronotype groups (all *p* < 0.05). The corresponding effect sizes were V = 0.424 for shift work, V = 0.206 for chronic disease, V = 0.290 for skipping meals, V = 0.231 for skipping breakfast, and V = 0.214 for BMI ≥ 25. Significant pairwise comparisons are shown in the post hoc column of Table 2.

### 3.4. Dietary Intake According to Chronotype

According to the 24 h dietary recall, evening chronotypes consumed an average of 1835 kcal/day, 87.66 g/day protein, 103.33 g/day fat, and 137.06 g/day carbohydrates. Intermediate chronotypes consumed 1879 kcal/day, 95.48 g/day protein, 110.97 g/day fat, and 123.50 g/day carbohydrates, while morning chronotypes consumed 1883 kcal/day, 89.52 g/day protein, 109.47 g/day fat, and 133.74 g/day carbohydrates. No statistically significant differences were observed in energy (*p* = 0.797), protein (*p* = 0.192), or fat intake (*p* = 0.178). However, carbohydrate intake differed significantly among chronotype groups (*p* < 0.001). Dietary fiber intake was also significantly different, with evening chronotypes having the lowest intake (14.13 g/day) compared with intermediate (15.88 g/day) and morning chronotypes (15.86 g/day) (*p* = 0.009).

### 3.5. Characteristics According to BMI Classification

BMI was classified according to World Health Organization criteria. Because the number of participants in the underweight category was insufficient for a separate statistical analysis, underweight and normal weight participants were combined into a single category. Age differed significantly across BMI classes (*p* = 0.003; η^2^ = 0.060). Bonferroni post hoc analysis showed that the mean age of the normal/underweight group was lower than both the overweight and obese groups (*p* = 0.036 and *p* = 0.006, respectively), but the difference was not statistically significant when comparing the overweight and obese groups (*p* = 1.000).

IPAQ MET-min/week differed significantly across BMI classes (*p* = 0.011; η^2^ = 0.047); post hoc analysis showed that the obese group had lower levels of physical activity compared to both the normal/underweight and overweight groups (*p* = 0.018 and *p* = 0.045, respectively), whereas there was no significant difference between the normal/underweight and overweight groups.

DEBQ restricted eating scores did not differ significantly between BMI classes (*p* = 0.092). However, emotional eating, external eating, total DEBQ scores, and total number of meals showed significant differences between BMI classes (all *p* < 0.05, Table 3). Bonferroni post hoc analysis showed that emotional eating was higher in the obese group compared to both normal/underweight and overweight groups (*p* < 0.001 and *p* = 0.007, respectively; η^2^ = 0.084). External eating was also higher in the obese group compared to the normal/underweight and overweight groups (*p* = 0.048 and *p* = 0.020, respectively; η^2^ = 0.043). The total DEBQ score was higher in the obese group compared to both the normal/underweight and overweight groups (*p* < 0.001 and *p* = 0.001, respectively; η^2^ = 0.104). The total number of meals was higher in the obese group compared to both the normal/underweight and overweight groups (*p* < 0.001 and *p* = 0.002, respectively; η^2^ = 0.161).

University or higher education level did not show a significant difference between BMI classes (84.1% in the normal/underweight group, 72.5% in the overweight group, and 69.2% in the obese group; *p* = 0.104). Among categorical variables, no significant difference was found between study schedule, skipping meals, and skipping breakfast and BMI classes (all *p* > 0.05). The presence of chronic disease showed a significant difference between BMI classes (*p* < 0.001; V = 0.487), with a higher rate in the obese group compared to both the normal/underweight and overweight groups in pairwise comparisons (both *p* < 0.001). Physical inactivity also showed a significant difference between BMI classes (*p* = 0.003; V = 0.251), with higher rates in the overweight and obese groups compared to the normal/underweight group (*p* = 0.005 and *p* = 0.011, respectively).

### 3.6. Characteristics According to Chronic Disease Status

The values for chronic disease and non-chronic disease groups according to the main study variables are summarized in Table 4. There was no significant difference in mean age according to chronic disease status (*p* = 0.209). Participants with chronic diseases were found to have significantly higher BMI, waist circumference, and hip circumference values compared to those without chronic diseases. BMI was 23.99 ± 3.01 kg/m^2^ in participants without chronic diseases, while it was 28.02 ± 4.70 kg/m^2^ in those with chronic diseases (*p* < 0.001; d = 1.121). Waist circumference was 77.87 ± 8.29 cm and 86.40 ± 11.93 cm, respectively (*p* < 0.001; d = 0.896), and hip circumference was 96.57 ± 7.29 cm and 104.11 ± 8.68 cm, respectively (*p* < 0.001; d = 0.975). MEQ score and IPAQ MET-minutes/week values did not differ significantly according to chronic disease status (*p* = 0.740 and *p* = 0.124, respectively).

When examining the DEBQ subscales, emotional eating and external eating scores were significantly higher in participants with chronic illness. The emotional eating score was 29.77 ± 12.84 in participants without chronic illness, while it was 34.74 ± 14.32 in those with chronic illness (*p* = 0.019; d = 0.374). The external eating score was 27.67 ± 5.78 and 31.40 ± 6.06, respectively (*p* < 0.001; d = 0.637). The total DEBQ score was also significantly higher in participants with chronic illness (95.40 ± 19.38 vs. 86.17 ± 17.89; *p* = 0.002; d = 0.503). The DEBQ restricted eating score did not show a significant difference according to chronic illness status (*p* = 0.603). Total number of meals was higher in participants with chronic illness (4.07 ± 0.82 vs. 3.62 ± 0.68; *p* < 0.001; d = 0.615).

When categorical variables were examined, shift work, skipping meals, skipping breakfast, and physical inactivity did not show a significant difference according to chronic disease status (all *p* > 0.05). In contrast, the proportion of participants with BMI ≥ 25 was 30.1% in those without chronic disease and 64.9% in those with chronic disease (*p* < 0.001; V = 0.325). Obesity prevalence was also higher in participants with chronic disease compared to those without chronic disease (38.6% vs. 3.0%; *p* < 0.001; V = 0.475).

### 3.7. Multivariable Regression Models

In the multivariable logistic regression model for breakfast skipping, shift work, MEQ score, DEBQ emotional eating score, and being a healthcare worker remained significant in the final model. Shift work was positively associated with breakfast skipping (B = 1.94; OR = 6.94; 95% CI: 2.57–18.72; *p* < 0.001), whereas higher MEQ score, higher DEBQ emotional eating score, and being a healthcare worker were negatively associated with breakfast skipping (Table 5). In the linear regression model for DEBQ emotional eating score, BMI was positively associated with emotional eating, whereas MEQ score and being a healthcare worker were negatively associated with emotional eating. In the linear BMI model, presence of chronic disease, age, IPAQ MET-min/week, DEBQ emotional eating, and healthcare worker status remained significant independent variables. Presence of chronic disease, older age, and higher DEBQ emotional eating score were positively associated with BMI, whereas higher physical activity and healthcare worker status were negatively associated with BMI. In the BMI ≥ 25 logistic regression model, presence of chronic disease, age, and DEBQ emotional eating remained significant independent variables. Presence of chronic disease was associated with higher odds of BMI ≥ 25 (OR = 3.84; 95% CI: 1.96–7.55; *p* < 0.001), age per 10 years was associated with higher odds of BMI ≥ 25 (OR = 2.03; 95% CI: 1.30–3.18; *p* = 0.002), and DEBQ emotional eating score was positively associated with BMI ≥ 25 (OR = 1.03; 95% CI: 1.01–1.06; *p* = 0.019).

## 4. Discussion

In the present study, marital status differed according to the study schedule, with a higher proportion of married participants in the fixed-schedule group. Furthermore, while education level did not show a significant difference between BMI classes in the present analysis, chronic disease status differed significantly between BMI classes. Although relevant findings have been reported in the previous literature, Efil’s [30] obesity prevalence study revealed higher obesity rates among married women, while Fouad et al. [31] reported a positive association between low education level and obesity [30,31].

A plethora of studies have hitherto been conducted in an attempt to ascertain the relationship between smoking and obesity. However, the results of these studies have been found to be incommensurate. In this study, smoking status did not differ significantly according to work schedule. In addition, the research conducted by Efil [30] and Çayır et al. [32] yielded similar findings, indicating an absence of a substantial correlation between smoking and obesity. This finding suggests that the relationship between smoking and obesity is not consistent in the extant literature [30,32].

Research conducted to date on the relationship between smoking and obesity has yielded conflicting results. In the present study, no statistically significant association was observed between smoking status and obesity-related outcomes. As indicated by the findings of Efil [30] and Çayır et al. [32], the relationship between smoking and obesity remains inconclusive.

Physical activity level did not differ significantly according to work schedule in the present study. Thus, the present findings do not directly support an association between shift work and lower physical activity, although previous studies have reported such relationships [33,34]. Furthermore, a multitude of studies have demonstrated that individuals engaged in shift work exhibit diminished levels of physical activity and reduced participation rates in recreational activities necessitating exercise or movement in comparison to those not engaged in shift work [35,36,37,38]. Consequently, although the effects of shift work and work environment factors on individuals’ physical activity habits and obesity status have yielded different results in various studies, it is generally thought that there may be a negative relationship.

In relation to eating behaviors, studies were established that emotional eating scores were elevated in obese individuals, and this consistency lends support to the notion of a strong association between emotional eating behaviors and obesity. Vasileiou et al. [39] reported that obese individuals exhibited higher emotional eating scores compared to those of normal weight, the robust correlation between obesity and emotional eating. Burgazlı [40] revealed that individuals with obesity demonstrated higher emotional eating scores in comparison to individuals of a normal weight, a distinction that was particularly evident among the female demographic. Furthermore, the study by Karanfil et al. [41] determined that obese individuals exhibited elevated emotional, restrictive, and external eating scores in comparison to individuals with a normal weight.

Recent research findings provide substantial evidence to support the notion of a strong correlation between obesity and emotional eating. The available evidence demonstrates that these behaviors can be triggered by the presence of stress, depression or anxiety, and that they can result in individuals consuming significantly more food than they would in a state of emotional equilibrium. Consequently, the observation that obese individuals demonstrate emotional eating habits with greater frequency is emphasized as a pivotal factor in weight management and treatment processes. The observed relationship between chronotype and eating behaviors is consistent with the findings of Arslan et al., Beaulieu et al., and Tüysüz, who reported that individuals with an evening chronotype exhibited higher levels of emotional, restrained, and external eating. The extant literature suggests that emotional eating behaviors may be triggered by emotional states, including stress, depression and anxiety, and that such behaviors can result in overeating. The current findings are generally consistent with this evidence, as evening chronotype is associated with higher restricted eating, emotional eating, external eating, and total DEBQ scores. Nevertheless, differences and similarities between these studies may also be due to methodological differences, differences in sample characteristics, or differences in measurement instruments [42,43,44].

With regard to dietary intake, individuals who were classified as evening chronotype were noted to exhibit elevated levels of carbohydrate consumption and reduced levels of fiber intake. However, it should be noted that certain discrepancies may be attributable to methodological variations, including variations in sample size and the distribution of chronotypes. In a study conducted by Çakır et al. on university students, the percentage of daily energy intake from carbohydrates and the percentage meeting the recommended amount were found to be lower in morning individuals than in evening chronotypes. The percentage of daily energy intake from fat and the percentage meeting the recommended amount were also found to be lower in evening chronotypes compared to morning chronotypes. However, no statistically significant difference was observed between the chronotypes of the students and their daily energy, fat, and carbohydrate intakes [45]. In a similar study, the proportion of total energy from fat in the diet was found to be statistically significantly higher in morning women compared to evening and intermediate chronotype women. These results may be explained by the tendency to consume more food during the late-night hours, as well as a preference for foods rich in carbohydrates. Furthermore, the tendency of evening chronotypes to consume their meals predominantly late in the evening and to exhibit a preference for packaged/processed food products is considered among the reasons for low fiber consumption [46].

This study shows that the evening chronotype is characterized by higher carbohydrate intake and lower dietary fiber intake, while total energy intake did not differ significantly between chronotype groups. The findings indicated that lower physical activity was associated with BMI classification, with lower IPAQ MET-min/week values and higher rates of physical inactivity observed in higher BMI classes. Additionally, emotional eating scores were found to be significantly higher in obese individuals.

Previous studies have reported that low physical activity levels are associated with higher body weight, BMI, and waist and hip measurements, while individuals with obesity tend to exhibit higher emotional eating scores. The literature also indicates that sociodemographic factors, including marital status and lower educational attainment, are associated with less favorable obesity-related anthropometric measurements, potentially due to differences in nutritional awareness and socioeconomic status. Furthermore, previous research has shown that shift workers are more likely to skip breakfast regularly and that irregular working hours may adversely affect meal regularity and overall eating habits [44,47,48].

Evening chronotype is associated with less healthy lifestyle patterns, such as higher energy intake, increased evening fast-food consumption, and lower fruit and vegetable intake. These individuals also tend to have lower fiber intake and higher carbohydrate intake, consistent with tendencies toward late-night eating and preference for energy-dense foods [45,46,49,50]. The participants in the present study had a mean body weight of 67.30 kg, a mean BMI of 25.20 kg/m^2^, a mean waist circumference of 80.43 cm, and a mean hip circumference of 98.83 cm (Table 2). Although previous studies have reported associations between evening chronotype and adverse anthropometric characteristics, no significant differences in body weight and BMI according to chronotype were identified in our study, and hip circumference was not higher in the evening-type group. Shift workers skip meals, especially breakfast, more frequently than fixed-day workers, reflecting the impact of irregular work schedules on eating patterns [25,51,52].

This study examined the variables of work schedule, chronotype, BMI classification, chronic disease status, physical activity, and eating behavior. In multivariate models, being a healthcare worker was negatively associated with skipping breakfast, emotional eating score, and BMI. Marital status differed according to work schedule. Education level did not show a significant difference among BMI classes. Lower physical activity was found to be associated with BMI classification. Finally, obese individuals had significantly higher emotional eating and external eating scores, while restrictive eating scores remained unchanged. Individuals with an evening chronotype had higher restrictive, emotional, external eating, and total DEBQ scores. In addition, data on skipping breakfast, in particular, indicated that the shift worker group was more prone to skipping breakfast. Our study emphasized the role of chronotype, work schedule, physical activity, and emotional state, in addition to energy intake, in the development of obesity.

The recommendations set out in the report include the development of nutrition and exercise programs that are tailored to evening chronotypes; the establishment of individual nutritional counseling and workplace policies for shift workers; and the implementation of awareness training to reduce the risk of obesity in individuals with low educational attainment. It is recommended that obese individuals receive psychological support and education on emotional eating control programs; that regular eating habits be established; that workplaces be equipped with healthy snacks and flexible meal times; that social and organizational support be provided to increase physical activity; that personalized interventions be implemented using multidisciplinary team approaches; that studies be continued in different demographic groups; and that behavioral interventions and training programs be planned.

## 5. Limitations

The limitations of this study primarily include the sample size and the focus on specific occupational groups and regional characteristics of the selected participants, which may restrict the generalizability of the results. Moreover, the subjective nature of the self-reported survey and nutritional records, in addition to the potential presence of bias, has the potential to influence the study’s findings. The cross-sectional design also has limitations in terms of its ability to identify changes over time and establish cause-and-effect relationships. The lack of a detailed analysis of genetic and environmental factors may be insufficient to fully explain variations in obesity and eating behaviors. It is important to note that the geographical and institutional limitations of the research may preclude the direct applicability of the results to different regions and populations.

## 6. Conclusions

In this study, no significant differences were observed in body weight or BMI across different chronotype groups. However, hip circumference measurements were found to be higher in the morning-type group compared to the intermediate-type group. The evening chronotype was found to be associated with higher emotional, restrained, and external eating scores.

The study’s findings indicate that, education level, anthropometric measurements, physical activity level, and eating habits were associated with BMI-related outcomes. Shift work was associated with meal skipping and breakfast skipping, but it was not associated with BMI ≥ 25 in the group comparison; education level did not differ significantly across BMI classes. Furthermore, emotional and external eating scores were higher in obese participants, restrained eating did not differ significantly, and lower physical activity was associated with BMI classification. In light of the aforementioned factors, it is evident that implementing bespoke lifestyle modifications, with consideration for individual characteristics such as emotional eating habits, age, and physical activity, is imperative in the endeavor to combat obesity, particularly among female professionals who work shifts. Consequently, a multidisciplinary and holistic approach that considers chronotype, work schedule, physical activity, and sociodemographic factors is imperative in obesity management. The recommendation is for future longitudinal studies to employ larger sample sizes and objective measurement methods.

## Figures and Tables

**Table 1:** Baseline characteristics and scale scores according to work schedule.

Variable	Fixed Schedule (n = 104)	Shift Work (n = 86)	*p*	Effect Size
**Age (years)**	35.34 ± 8.84	30.85 ± 5.66	<0.001 ^c^	d = 0.593
**BMI (kg/m^2^)**	25.61 ± 3.65	24.71 ± 4.42	0.126 ^c^	d = 0.224
**Waist circumference (cm)**	81.76 ± 9.82	78.83 ± 10.64	0.050 ^c^	d = 0.288
**Hip circumference (cm)**	100.31 ± 7.48	97.03 ± 9.22	0.009 ^c^	d = 0.394
**MEQ score**	55.74 ± 10.11	44.92 ± 11.24	<0.001 ^c^	d = 1.017
**IPAQ MET-min/week**	1479.03 ± 985.26	1335.31 ± 1033.15	0.329 ^c^	d = 0.143
**DEBQ emotional eating**	31.59 ± 12.93	30.86 ± 14.13	0.712 ^c^	d = 0.054
**DEBQ external eating**	25.92 ± 5.95	32.26 ± 4.19	<0.001 ^c^	d = 1.211
**DEBQ total score**	86.37 ± 20.96	92.06 ± 15.30	0.032 ^c^	d = 0.306
**Married**	56 (53.8)	34 (39.5)	0.049 ^a^	V = 0.143
**University or higher education**	72 (69.2)	78 (90.7)	<0.001 ^a^	V = 0.262
**Healthcare worker**	18 (17.3)	62 (72.1)	<0.001 ^a^	V = 0.552
**Presence of chronic disease**	31 (29.8)	26 (30.2)	0.949 ^a^	V = 0.005
**Smoking**	55 (52.9)	43 (50.0)	0.692 ^a^	V = 0.029
**Meal skipping**	62 (59.6)	67 (77.9)	0.007 ^a^	V = 0.195
**Breakfast skipping**	17 (16.3)	44 (51.2)	<0.001 ^a^	V = 0.371
**Physical inactivity**	28 (26.9)	21 (24.4)	0.694 ^a^	V = 0.028
**Overweight/obese (BMI ≥ 25)**	44 (42.3)	33 (38.4)	0.582 ^a^	V = 0.040

Continuous variables are presented as mean ± SD according to distribution; categorical variables are presented as n (%). Superscripts for *p* values: ^a^ Pearson chi-square test; ^c^ independent-samples *t*-test; Cohen’s d for independent-samples *t*-tests. Effect sizes are reported for the primary comparisons and associations irrespective of statistical significance. *p* < 0.05 was considered statistically significant.

**Table 2:** Anthropometric, behavioral, and scale variables according to chronotype classification.

Variable	Morning Type (n = 50)	Intermediate Type (n = 86)	Evening Type (n = 54)	*p*	Effect Size	Post Hoc
**Age (years)**	37.18 ± 8.77	32.33 ± 7.19	31.28 ± 6.85	<0.001 ^e^	η^2^ = 0.090	1 > 2; 1 > 3
**Body weight (kg)**	68.65 (59.12–74.07)	62.20 (59.02–71.00)	66.90 (58.00–75.00)	0.186 ^f^	ε^2^ = 0.007	-
**BMI (kg/m^2^)**	25.71 ± 3.54	24.54 ± 3.48	25.77 ± 5.08	0.126 ^e^	η^2^ = 0.022	-
**Hip circumference (cm)**	101.63 ± 7.91	96.78 ± 7.72	99.50 ± 9.31	0.004 ^e^	η^2^ = 0.058	1 > 2
**IPAQ MET-min/week**	1282.38 ± 984.63	1643.98 ± 1016.00	1169.53 ± 949.66	0.013 ^e^	η^2^ = 0.045	2 > 3
**DEBQ restrained eating**	26.74 ± 5.80	28.03 ± 6.37	32.26 ± 7.31	<0.001 ^e^	η^2^ = 0.103	3 > 1; 3 > 2
**DEBQ emotional eating**	28.12 ± 10.50	29.21 ± 12.08	37.43 ± 15.96	<0.001 ^e^	η^2^ = 0.085	3 > 1; 3 > 2
**DEBQ external eating**	26.44 ± 7.91	29.34 ± 5.13	30.09 ± 5.01	0.005 ^e^	η^2^ = 0.056	2 > 1; 3 > 1
**DEBQ total score**	81.30 ± 17.71	86.58 ± 16.08	99.78 ± 19.23	<0.001 ^e^	η^2^ = 0.146	3 > 1; 3 > 2
**Total number of meals**	3.84 ± 0.47	3.59 ± 0.60	3.94 ± 1.07	0.017 ^e^	η^2^ = 0.043	1 > 2; 3 > 2
**Shift work**	10 (20.0)	35 (40.7)	41 (75.9)	<0.001 ^a^	V = 0.424	2 > 1; 3 > 1; 3 > 2
**Presence of chronic** **disease**	22 (44.0)	18 (20.9)	17 (31.5)	0.017 ^a^	V = 0.206	1 > 2
**Meal skipping**	32 (64.0)	49 (57.0)	48 (88.9)	<0.001 ^a^	V = 0.290	3 > 1; 3 > 2
**Breakfast skipping**	10 (20.0)	25 (29.1)	26 (48.1)	0.006 ^a^	V = 0.231	3 > 1
**Physical inactivity**	17 (34.0)	15 (17.4)	17 (31.5)	0.055 ^a^	V = 0.175	-
**Overweight/obese** **(BMI ≥ 25)**	26 (52.0)	25 (29.1)	26 (48.1)	0.013 ^a^	V = 0.214	1 > 2

Continuous variables are presented as mean ± SD or median (Q1–Q3) according to distribution; categorical variables are presented as n (%). Superscripts for *p* values: ^a^ Pearson chi-square test; ^e^ one-way ANOVA with Bonferroni-adjusted pairwise comparisons when the overall test was significant; ^f^ Kruskal–Wallis test with Dunn–Bonferroni pairwise comparisons when the overall test was significant. Bonferroni-adjusted pairwise comparisons of column proportions were used for significant categorical variables. Effect-size metrics: Cramer’s V for chi-square tests, η^2^ for one-way ANOVA, and ε^2^ for Kruskal–Wallis tests. Effect sizes are reported for the primary comparisons and associations irrespective of statistical significance. Only significant pairwise comparisons are shown in the post hoc column. For Table 2, groups were coded as 1 = morning type, 2 = intermediate type, and 3 = evening type. *p* < 0.05 was considered statistically significant.

**Table 3:** Main variables according to BMI classification.

Variable	Normal/ Underweight (n = 113)	Overweight (n = 51)	Obese (n = 26)	*p*	Effect Size	Post Hoc
**Age (years)**	31.78 ± 6.69	34.96 ± 8.82	36.69 ± 9.17	0.003 ^e^	η^2^ = 0.060	2 > 1; 3 > 1
**MEQ score**	51.48 ± 11.78	50.86 ± 12.22	48.04 ± 11.89	0.416 ^e^	η^2^ = 0.009	-
**IPAQ MET-min/week**	1525.77 ± 1010.69	1442.82 ± 1048.13	871.56 ± 727.93	0.011 ^e^	η^2^ = 0.047	1 > 3; 2 > 3
**DEBQ restrained eating**	28.12 ± 7.38	29.41 ± 5.63	31.23 ± 6.07	0.092 ^e^	η^2^ = 0.025	-
**DEBQ emotional eating**	29.17 ± 11.83	31.00 ± 14.33	40.85 ± 14.67	<0.001 ^e^	η^2^ = 0.084	3 > 1; 3 > 2
**DEBQ external eating**	28.54 ± 5.69	27.78 ± 7.16	31.85 ± 4.66	0.017 ^e^	η^2^ = 0.043	3 > 1; 3 > 2
**DEBQ total score**	85.83 ± 16.69	88.20 ± 19.88	103.92 ± 18.75	<0.001 ^e^	η^2^ = 0.104	3 > 1; 3 > 2
**Total number of meals**	3.57 ± 0.69	3.82 ± 0.59	4.46 ± 0.86	<0.001 ^e^	η^2^ = 0.161	3 > 1; 3 > 2
**University or higher** **education**	95 (84.1)	37 (72.5)	18 (69.2)	0.104 ^a^	V = 0.154	-
**Shift work**	53 (46.9)	20 (39.2)	13 (50.0)	0.574 ^a^	V = 0.076	-
**Presence of chronic disease**	20 (17.7)	15 (29.4)	22 (84.6)	<0.001 ^a^	V = 0.487	3 > 1; 3 > 2
**Meal skipping**	70 (61.9)	37 (72.5)	22 (84.6)	0.059 ^a^	V = 0.173	-
**Breakfast skipping**	34 (30.1)	20 (39.2)	7 (26.9)	0.424 ^a^	V = 0.095	-
**Physical inactivity**	19 (16.8)	19 (37.3)	11 (42.3)	0.003 ^a^	V = 0.251	2 > 1; 3 > 1

Continuous variables are presented as mean ± SD according to distribution; categorical variables are presented as n (%). Superscripts for *p* values: ^a^ Pearson chi-square test; ^e^ one-way ANOVA with Bonferroni-adjusted pairwise comparisons when the overall test was significant. Bonferroni-adjusted pairwise comparisons of column proportions were used for significant categorical variables. Effect-size metrics: Cramer’s V for chi-square tests and η^2^ for one-way ANOVA. Effect sizes are reported for the primary comparisons and associations irrespective of statistical significance. Only significant pairwise comparisons are shown in the post hoc column. For Table 3, groups were coded as 1 = normal/underweight, 2 = overweight, and 3 = obese. *p* < 0.05 was considered statistically significant.

**Table 4:** Main study variables according to chronic disease status.

Variable	No Chronic Disease (n = 133)	Chronic Disease Present (n = 57)	*p*	Effect Size
**Age (years)**	32.83 ± 7.41	34.40 ± 8.84	0.209 ^c^	d = 0.200
**BMI (kg/m^2^)**	23.99 ± 3.01	28.02 ± 4.70	<0.001 ^c^	d = 1.121
**Waist circumference (cm)**	77.87 ± 8.29	86.40 ± 11.93	<0.001 ^c^	d = 0.896
**Hip circumference (cm)**	96.57 ± 7.29	104.11 ± 8.68	<0.001 ^c^	d = 0.975
**MEQ score**	50.65 ± 12.00	51.28 ± 11.76	0.740 ^c^	d = 0.053
**IPAQ MET-min/week**	1487.56 ± 995.22	1242.29 ± 1022.49	0.124 ^c^	d = 0.244
**DEBQ restrained eating**	28.74 ± 7.18	29.26 ± 6.00	0.603 ^c^	d = 0.077
**DEBQ emotional eating**	29.77 ± 12.84	34.74 ± 14.32	0.019 ^c^	d = 0.374
**DEBQ external eating**	27.67 ± 5.78	31.40 ± 6.06	<0.001 ^c^	d = 0.637
**DEBQ total score**	86.17 ± 17.89	95.40 ± 19.38	0.002 ^c^	d = 0.503
**Total number of meals**	3.62 ± 0.68	4.07 ± 0.82	<0.001 ^c^	d = 0.615
**Shift work**	60 (45.1)	26 (45.6)	0.949 ^a^	V = 0.005
**Meal skipping**	86 (64.7)	43 (75.4)	0.145 ^a^	V = 0.106
**Breakfast skipping**	43 (32.3)	18 (31.6)	0.919 ^a^	V = 0.007
**Physical inactivity**	31 (23.3)	18 (31.6)	0.232 ^a^	V = 0.087
**Overweight/obese (BMI ≥ 25)**	40 (30.1)	37 (64.9)	<0.001 ^a^	V = 0.325
**Obesity (BMI ≥ 30)**	4 (3.0)	22 (38.6)	<0.001 ^a^	V = 0.475

Continuous variables are presented as mean ± SD according to distribution; categorical variables are presented as n (%). Superscripts for *p* values: ^a^ Pearson chi-square test; ^c^ independent-samples *t*-test. Effect-size metrics: Cramer’s V for chi-square tests and Cohen’s d for independent-samples *t*-tests. Effect sizes are reported for the primary comparisons and associations irrespective of statistical significance. *p* < 0.05 was considered statistically significant.

**Table 5:** Multivariable regression models for the main outcomes.

Model	Independent Variable	B	OR/β	95% CI	*p*
**Model 1: Breakfast skipping**	**Shift work**	1.94	6.94	2.57–18.72	<0.001 ^j^
	**MEQ score**	−0.07	0.93	0.89–0.96	<0.001 ^j^
	**DEBQ emotional eating**	−0.09	0.92	0.88–0.96	<0.001 ^j^
	**Healthcare worker**	−1.43	0.24	0.10–0.58	0.002 ^j^
**Model 2: DEBQ emotional eating score**	**BMI**	0.84	0.25	0.41–1.27	<0.001 ^k^
	**MEQ score**	−0.30	−0.27	−0.45–−0.16	<0.001 ^k^
	**Healthcare worker**	−9.36	−0.34	−13.00–−5.72	<0.001 ^k^
**Model 3: BMI**	**Presence of chronic disease**	3.27	0.37	2.14–4.41	<0.001 ^k^
	**Age (10 years)**	0.94	0.18	0.27–1.62	0.006 ^k^
	**IPAQ MET-min/week (500 units)**	−0.28	−0.14	−0.54–−0.02	0.036 ^k^
	**DEBQ emotional eating**	0.08	0.28	0.04–0.12	<0.001 ^k^
	**Healthcare worker**	−1.12	−0.14	−2.12–−0.12	0.029 ^k^
**Model 4: BMI ≥25**	**Presence of chronic disease**	1.35	3.84	1.96–7.55	<0.001 ^j^
	**Age (10 years)**	0.71	2.03	1.30–3.18	0.002 ^j^
	**DEBQ emotional eating**	0.03	1.03	1.01–1.06	0.019 ^j^

For logistic regression models (Models 1 and 4), OR and 95% CI are reported; for linear regression models (Models 2 and 3), standardized β and the 95% CI for the unstandardized B coefficient are reported. Superscripts: ^j^ multivariable logistic regression, Wald test, robust SE; ^k^ multiple linear regression, robust SE. *p* < 0.05 was considered statistically significant. Model selection. For each dependent variable, candidate variables were first identified using univariable analyses at *p* < 0.05. Only variables that remained significant at *p* < 0.05 in the multivariable model were retained. Model summaries: breakfast skipping n = 190/events = 61, McFadden R^2^ = 0.272; emotional eating R^2^ = 0.271; BMI R^2^ = 0.370; BMI ≥ 25 n = 190/events = 77, McFadden R^2^ = 0.137.

## Data Availability

The data supporting the findings of this study are available from the corresponding author upon reasonable request. The thesis from which this article was derived will be publicly accessible through the National Thesis Center of the Council of Higher Education (YÖK), Türkiye.
